# Biosafety and potency of high-molecular-weight hyaluronic acid with intratympanic dexamethasone delivery for acute hearing loss

**DOI:** 10.3389/fphar.2024.1294657

**Published:** 2024-01-16

**Authors:** Yu-Jung Hwang, Seung Ha Oh, Jun Ho Lee, Moo Kyun Park, Myung-Whan Suh

**Affiliations:** ^1^ Department of Otorhinolaryngology-Head and Neck Surgery, Seoul National University Hospital, Seoul, Republic of Korea; ^2^ Sensory Organ Research Institute, Seoul National University Medical Research Center, Seoul, Republic of Korea

**Keywords:** drug delivery, ototoxic hearing loss, intratympanic injection, hyaluronic acid, high performance liquid chromatography

## Abstract

**Objective:** This study evaluated the potential of high-molecular-weight hyaluronic acid (HHA) as an intratympanic (IT) drug delivery vehicle for dexamethasone (D) in treating acute hearing loss. We compared the efficacy, safety, and residence time of HHA to the standard-of-care IT drug delivery method.

**Methods:** Endoscopic examinations were used to track tympanic membrane (TM) healing post-IT injection. Micro-computed tomography (CT) was used to gauge drug/vehicle persistence in the bulla air space. Histological analyses covered the middle ear, TM, and hair cell counts. Auditory brainstem responses (ABR) were used to measure hearing thresholds, while high-performance liquid chromatography (HPLC) was employed to quantify cochlear perilymph dexamethasone concentrations.

**Results:** The HHA + D group had a notably prolonged drug/vehicle residence time in the bulla (41 ± 27 days) compared to the saline + D group (1.1 ± 0.3 days). Complete TM healing occurred without adverse effects. Histology revealed no significant intergroup differences or adverse outcomes. Hearing recovery trends favored the HHA + D group, with 85.0% of ears showing clinically meaningful improvement. D concentrations in cochlear perilymph were roughly double in the HHA group.

**Conclusion:** HHA is a promising vehicle for IT drug delivery in treating acute hearing loss. It ensures extended residence time, augmented drug concentrations in targeted tissues, and safety. These results highlight the potential for HHA + D to excel beyond existing standard-of-care treatments for acute hearing loss.

## Introduction

Acute-onset hearing loss can be effectively treated with steroids. Systemic steroid therapy has been the predominant treatment for such hearing loss, with the belief that a higher steroid concentration correlates with better treatment outcomes. Studies have demonstrated the efficacy of steroids in treating acute hearing loss from causes such as Ménière’s disease (Cohn and Salomon, 2005; Garduno-Anaya et al., 2005; Hyun et al., 2006; Zhao et al., 2007; McCall et al., 2010), acoustic trauma (Zhou et al., 2013), and ototoxic medication (Hill et al., 2008). However, large systemic steroid doses can lead to unwanted side effects (Min et al., 2012). Furthermore, the blood–labyrinth barrier makes it challenging to maintain adequate steroid levels in the cochlea through systemic delivery.

Alternative local delivery approaches, such as intratympanic (IT) injection of dexamethasone (D), have been explored. Due to direct absorption via the round window membrane, IT injections achieve higher steroid concentrations in the perilymph compared to systemic routes (Bird et al., 2007; Bird et al., 2011). A systematic review indicated that IT steroids can benefit patients with acute SNHL who either cannot tolerate systemic therapy or show no response (Seggas et al., 2011; Wang et al., 2023). The American Academy of Otolaryngology–Head and Neck Surgery recommends IT steroid perfusion for patients with incomplete recovery from sudden sensorineural hearing loss (Chandrasekhar et al., 2019). Moreover, IT steroid injections have shown efficacy in treating acute ototoxic hearing loss (Fernandez et al., 2016) and acute acoustic trauma (Park et al., 2020).

However, the challenge with the current IT steroid method is the short residence time of the drug in the middle ear, limiting sustained therapeutic effects. It is estimated that the residence time of the current IT steroid is under several hours (Shim, 2016). An ideal delivery vehicle should resist drainage through the eustachian tube, deliver high drug concentrations to the cochlea, be biocompatible, be biodegradable, and be easily administered via IT injection. While various biomaterials have been examined as potential IT drug-delivery vehicles, many fail to meet these criteria (Park et al., 2020).

**FIGURE 1:**
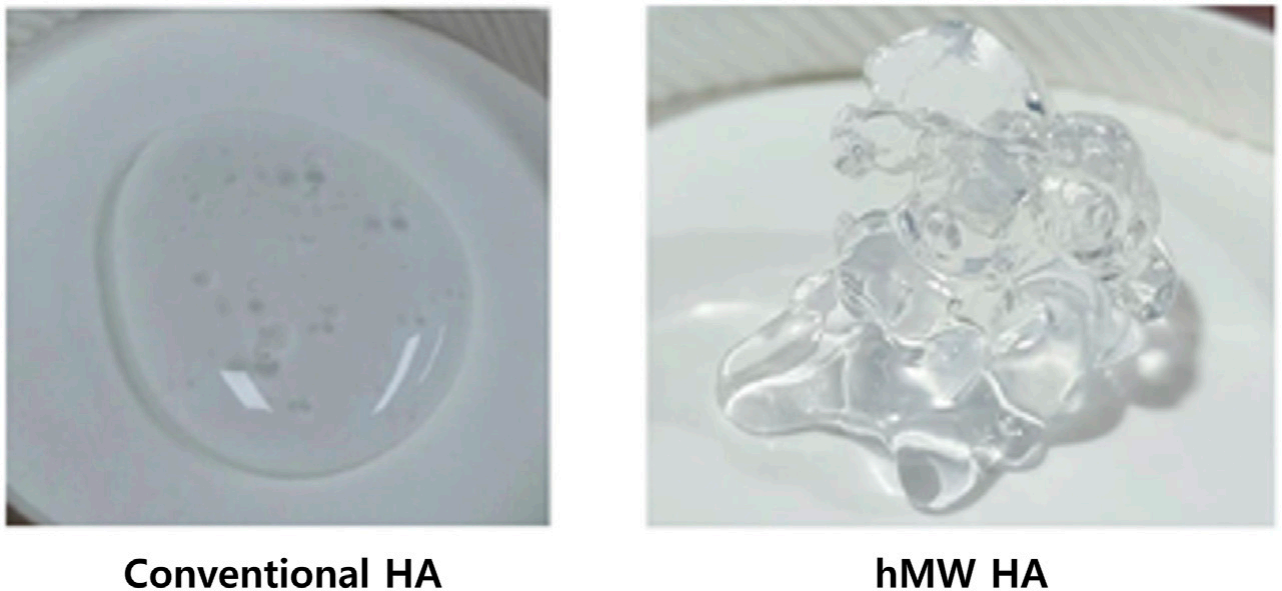
Gross morphology of HHA on a dish. HHA (right ) exhibits greater viscosity and adherence due to its significant molecular size (molecular weight of 5–10 MDa), compared to conventional hyaluronic acid (left) which is more fluidic (molecular weight of ≤3 MDa).

High-molecular-weight hyaluronic acid (HHA), a biogel with significant viscosity and adherence due to its large size (molecular weight of 5–10 MDa), stands out. Compared to conventional HA (molecular weight ≤3 MDa), HHA is more robust and adhesive (Figure 1). Prior research has highlighted the differences in physical properties and 9.6T MR imaging-based pharmacokinetics between HHA and conventional HA (Hwang et al., 2021). HHA demonstrated the ability to deliver higher gadolinium concentrations to the cochlea over an extended period without adverse effects. In this study, we assessed the therapeutic effect of D for treating acute hearing loss using HHA as the IT drug-delivery vehicle. We hypothesized that the HHA vehicle for IT D would yield superior outcomes in terms of hearing and cochlear perilymph drug concentrations compared to conventional IT D, which uses saline as the delivery vehicle.

## Materials and methods

### Experimental animals and drug/vehicle preparation

The animal preparation and experimental schedule closely followed a prior study (Li et al., 2021), albeit with different drugs and vehicles. This study received approval from the Animal Research Committee of Seoul National University Hospital. All animal care followed the guidelines set by the Institutional Animal Care and Use Committee Institute (IACUC 17–0217-C1A0 and IACUC 20-0149-S1A0). We used 26 male Sprague Dawley rats (52 ears; 6 weeks old, weighing 130–190 g). The ears were divided into three treatment groups: saline + D group (n = 17), HHA + D group (n = 20), and the hearing loss (HL) control group (n = 12). The D concentration (10 mg/mL) remained consistent between the saline + D and HHA + D groups. The HL control group underwent ototoxic hearing loss induction as in the study groups but did not receive any treatment. To minimize individual animal-associated variability, both the left and right ears of each rat were allocated appropriately.

The specific drug/vehicle composition and its preparation process are detailed in another report (Dae-Jung et al., 2020). Briefly, D disodium phosphate (a water-soluble salt form) was constituted in 10 mM phosphate buffer at 1.2% (w/v) concentration. HHA (5–10 million Da, MNH Bio Co. Ltd., Hwaseong-si, South Korea) was prepared at a 2.0% (w/v) concentration. To stave off bacterial and mold contamination, the HA underwent filtration through a 0.2 µm cellulose acetate membrane.

### IT drug delivery

Prior to inducing ototoxic hearing loss, rats underwent IT drug/vehicle administration. For anesthesia, we used intramuscular injections of xylazine (2 mL/kg; Rompun, Bayer, Leverkusen, Germany) and zoletil (1.2 mL/kg; Zoletil 50, Virbac, Carros, France). The IT drug/vehicle application employed a 1 cc syringe (KovaxSyringe 1 mL, South Korea Vaccine Co., Seoul, South Korea), paired with a 24G catheter (Angiocath Plus, BD, Sandy Utah, United States) and a mini extension tube (Mini-Volume Line, Insung Medical, Seoul, South Korea). This procedure, visualized under a surgical microscope (OPMI Pico, Carl-Zeiss, Oberkochen, Germany), began with creating an air vent in the anterior superior quadrant of the tympanic membrane (TM). The drug/vehicle was meticulously injected into the posterior superior quadrant at a slow rate (∼10 μL/s). The procedure was stopped either when the drug/vehicle filled the middle ear (bulla) entirely or upon leakage through the air vent. The HHA + D group and saline + D group had comparable injected volumes (0.05 ± 0.01 mL). Successful injections, representing 98.6% of the cases, were included in the study. Following one ear’s drug/vehicle injection, the other ear received an alternate drug/vehicle almost immediately, with an average time gap of 1.6 ± 0.6 min between injections. To counteract positional effects, rats were consistently placed in a straight prone position without tilting to either side until the study’s end. The type and sequence of drug/vehicle injections were randomized.

### Induction of ototoxic hearing loss

To induce ototoxic hearing loss, we administered cisplatin (2 mg/kg), gentamycin (120 mg/kg), and furosemide (90 mg/kg) intravenously over two consecutive days. Following anesthesia, the ototoxic drugs were slowly injected (0.18 ± 0.03 mL/min) into the vein on the lateral side of the tail using an Angiocath Plus 24-gauge needle. Ototoxic hearing loss induction occurred on the fourth day after IT drug delivery. The final day of this induction was labeled post-hearing loss day (PHD) 0.

### TM endoscopy and micro-computed tomography (CT)

An endoscope (GD-060, Chammed, Gunpo, South Korea) with a 2.7 mm diameter was paired with a smartphone (iPhone 4, Apple Inc., Cupertino, CA, United States) to capture images of the rats’ external auditory canal and TM. Observations were made for signs of inflammation, swelling, congestion, perforation, or other adverse effects. The surface integrity, perforation healing, and transparency of the TM were also evaluated. A micro-CT system (NFR PolarisG90; Nanofocusray Co., Ltd., Jeonju, South Korea) was used to assess the remaining drug/vehicle in the bulla. Both TM endoscopy and micro-CT procedures were conducted prior to and at 1 h after IT drug delivery, and subsequently on days 1, 4, 8, 12, 30, 45, and 60. Following drainage through the eustachian tube, the drug/vehicle was primarily detected at the base of the bulla (BB) and between the ossicle and cochlea. The final day where residual drug/vehicle was observable in these anatomical areas was termed the residence time (in days).

### Assessment of the hearing threshold via the auditory brainstem responses

The Smart EP system (Intelligent Hearing Systems, Miami, FL, United States) was utilized to evaluate hearing outcomes. Auditory brainstem responses (ABR) threshold tests were executed at frequencies of 8, 16, and 32 kHz within a soundproof chamber. Before ABR measurements, rats were anesthetized, as previously described. Subdermal needle electrodes were placed at the vertex (active electrode) and behind both the ipsilateral (reference electrode) and contralateral ears (ground electrode). The speaker was positioned in line with the external auditory canal, and the earphone tube was gently inserted into the ear canal. Hearing thresholds were deduced by identifying the minimum stimulus level eliciting clear III/V and SN10 waves. ABR testing was initiated at 90 dB SPL, with a reduction by 5 dB SPLeach iteration. ABR assessments coincided with the TM endoscopy and micro-CT measurements. A clinically significant enhancement in hearing was defined as a reduction of 15 dB SPL or more in the hearing threshold. Then we determined and compared the percentage of ears exhibiting a clinically notable improvement in hearing across groups.

### Middle ear histology

On the final day of the experiment (PHD 60), the middle ear was harvested and perfused with a solution of 4% paraformaldehyde and phosphate-buffered saline (PBS). Then it was fixed with 4% paraformaldehyde for 12 h at room temperature. Following decalcification using 0.1 M ethylenediaminetetraacetic acid (pH 7.4) for a period of 4 weeks, the bullae were embedded in paraffin wax. Then these were sectioned into 5 µm thick slices and stained with hematoxylin and eosin (H&E). In addition, Masson’s trichrome (MT) staining was employed to analyze the histological features of the connective tissue, such as collagen, collagen fibers, fibrin, muscles, and erythrocytes. The position of the TM was consistently identified by pinpointing the malleus head and its fibrous linkage to the TM. Then the mucosa at the BB was assessed. Using a light microscope (CX31, Olympus, Tokyo, Japan), measurements of the TM and BB mucosa thickness were taken using the DP2-BSW software (Olympus).

### Organ of Corti surface preparation and hair cell count

On the experiment’s concluding day, animals were anesthetized, after which the cochleae were extracted and perfused with 4% paraformaldehyde in PBS. Then these were fixed with 4% paraformaldehyde at 4°C for 24 h. The surface preparation of the Organ of Corti was carried out under a stereoscopic microscope (SZX7, Olympus, Tokyo, Japan). The cochlea was treated with a phalloidin stain (Alexa Fluor 546, Life Technologies, Oregon, United States) and subsequently rinsed three times with PBS for 5 min each time. Hair cells were visualized through z-stacking using a confocal microscope (Leica TCS SP8, Leica Microsystems, Wetzlar, Germany). Within a span of 200 μm in each turn, three rows of outer hair cells and inner hair cells were counted. For each turn, two distinct samples were utilized for the measurements. If the photomicrographs showed that the hair cells were missing or vacant, they were categorized as non-living.

### Pharmacokinetics from cochlear perilymph

Albino Hartley guinea pigs (weighing 300–350 g) were used for the high-performance liquid chromatography (HPLC) analysis of D. All of the procedures for preparing the drug/vehicle and IT drug-delivery injection were consistent with previously described methods. The only distinction was the injection volume due to the larger bulla size in guinea pigs compared to rats. The injection volume of the drug/vehicle was 0.10 ± 0.07 mL.

Perilymph sampling occurred <2 h after the IT D injection. Only ears with successful drug administration were considered for analysis. Prior to sampling, the hair behind the ear was shaved using shaving cream and a razor. A round cutting burr opened the bulla and revealed the round window. Carefully avoiding any damage to the round window membrane, bone dust and tissue fluid were removed with soft gauze from the bulla and the round window niche. A long-reach pipette tip (10 µL Long Reach Pipette Tip, Multimax, Salt Lake City, United States) was gently inserted into the round window to slowly sample 2 µL perilymph using a micropipette (Eppendorf Research plus, Eppendorf, Hamburg, Germany).

In total, 49 perilymph samples were collected and diluted 15 times with saline (28 µL) from the HHA + D group, saline + D group, and HL control group. Positive and negative control samples were prepared by diluting the standard stock solution of D sodium phosphate for quality assurance: 8 samples with a concentration of D of 10–2,500 ng/mL. HPLC analyses were conducted using the Agilent 1,260 infinity (Agilent, Palo Alto, CA, United States), while MS/MS analyses utilized the API 4000QTRAP (Absciex, Foster City, CA, United States).

The detailed method for MS/MS analysis can be found in Zhang et al. (2011). In summary, a standard curve was established by plotting the ratio of the standard peak area to the internal standard peak area, commonly referred to as the area ratio. For perilymph samples, the peak area was divided by the internal standard peak area to derive the area ratio. Then this ratio was used to determine concentration values on the curve. The concentration of D sodium phosphate was ascertained by comparing the area ratio between D sodium phosphate and the internal standard from the obtained chromatogram using a calibration curve.

### Statistical analyses

All statistical evaluations were conducted using the SPSS software (version 25.0; SPSS Inc., IBM Corp., Armonk, NY, United States). Continuous variables are presented as means ± standard deviations (SDs). The Mann–Whitney *U*-test was employed to compare the results across groups. *p*-values <0.05 were deemed statistically significant.

## Results

### Endoscopic examination of the TM

**FIGURE 2:**
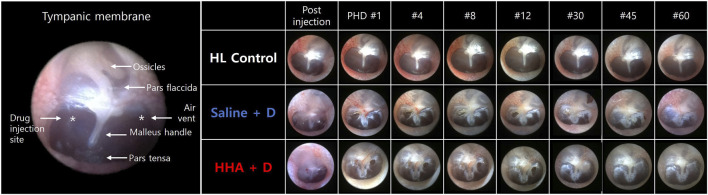
Endoscopic observations of the TM. Perforations created during injection healed within 20–26 days. No animals displayed residual TM perforation after 60 days. Perforation closure was observed 22 ± 13 days post-IT injection in the saline + D group and 21 ± 12 days in the high-molecular-weight hyaluronic acid HHA + D group. No animals exhibited adverse effects such as infection, inflammation, hyperemia, bulging, draining, or permanent perforation.

The TM perforations created during the IT injections healed effectively in all animals by the 60th day (Figure 2). Complete healing was observed at 22 ± 13 days post IT injection in the saline + D group, and 21 ± 12 days in the HHA + D group. The healing durations for perforations were comparable between the two groups (*p* = 0.549). The period of perforation closure in the HHA + D group was similar to that of the conventional HA + D group (Supplementary Figure S1). Because there were no IT interventions conducted in the HL control group, no perforations were present in their TMs. None of the animals (0%) exhibited adverse effects such as infection, inflammation, hyperemia, bulging, draining, or permanent perforation.

### Micro-CT of the bulla air space

**FIGURE 3:**
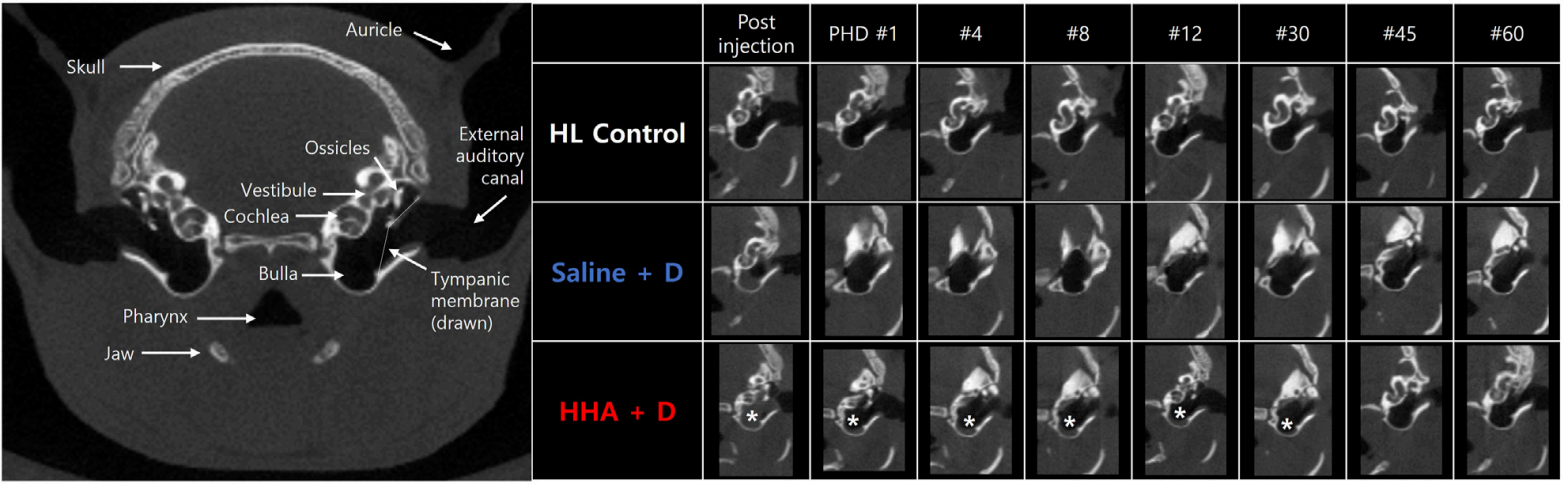
Micro-CT imaging of the bulla. Soft tissue density, indicative of vehicle/drug presence, was identified in the bulla. Persistence of this signal was monitored. In the saline + D group, the vehicle/drug remained in the bulla for 1.1 ± 0.3 days, whereas in the HHA + D group, it persisted for 41 ± 27 days. The observed duration in the bulla was notably longer in the HHA + D group than in the saline + D group. The asterisk denotes the soft tissue density signal in the CT image (indicative of residual vehicle/drug).

In the initial 5 days, the discernible volume of the drug/vehicle declined rapidly in both experimental groups (Figure 3). However, trace amounts were still detectable at the BB and between the ossicle and cochlea in the HHA + D group. The drug/vehicle persisted in the bulla for 1.1 ± 0.3 days in the saline + D group, and for 41 ± 27 days in the HHA + D group (Figure 3). Thus, the residence time was markedly longer in the HHA + D group (*p* < 0.001). And the duration of the vehicle/drug in the conventional HA + D group was 1.8 ± 2.4 days. In comparison, it was confirmed that the vehicle/drug in the HHA + D group persisted for a significantly longer period (Supplementary Figure S2). Because the HL control group did not receive any IT injections, no drug/vehicle was evident. Based on a prior publication, renewed visibility of volume in CT was suggestive of infection and/or inflammation. However, in this study, none of the animals showed signs of infection and/or inflammation as determined by micro-CT.

### Histology of the middle ear and hair cell

**FIGURE 4:**
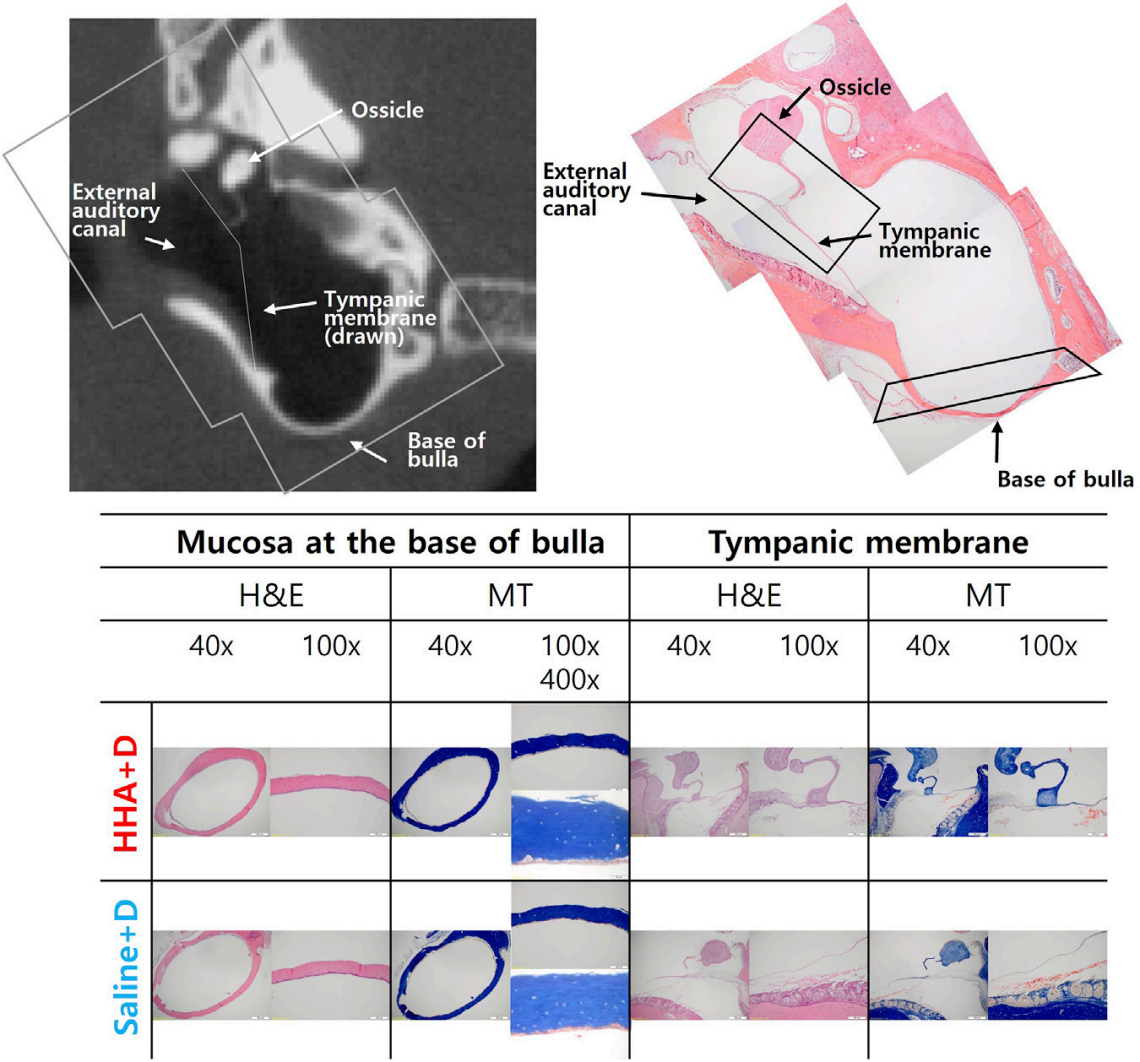
Middle ear histology. Mucosal histology at the bulla’s base showed similarity between the saline + D and HHA + D groups when stained with hematoxylin and eosin (H&E). No signs of inflammatory cell infiltration or mucosal thickening were evident under both low-power (40×) and high-power (×400) magnification. The thickness of the tympanic membrane was consistent between groups. Collagen and fibrin composition patterns, assessed via MT staining, also exhibited no discernible differences.

Histologically, no discernible differences were observed between the saline + D group and the HHA + D group. Specifically, the histological morphology of the mucosa at the BB was consistent across both groups. The thickness of the TM was comparable between the groups, measuring 9 µm in the saline + D group and 14 µm in the HHA + D group. Similarly, the mucosa thickness at the BB registered as 11 ± 5 µm in the saline + D group and 12 ± 9 µm in the HHA + D group (Figure 4). H&E staining did not reveal signs of inflammation or swelling. Moreover, after conducting MT staining, the patterns of collagen and fibrin composition appeared nearly indistinguishable between the two groups. Both groups showed an absence of connective tissue edema or hyperplasia. These findings in the HHA + D group demonstrated biosafety similar to the results observed in the previously conducted conventional HA + D group (Supplementary Figure S3).

### Organ of Corti surface preparation and hair cell count

**FIGURE 5:**
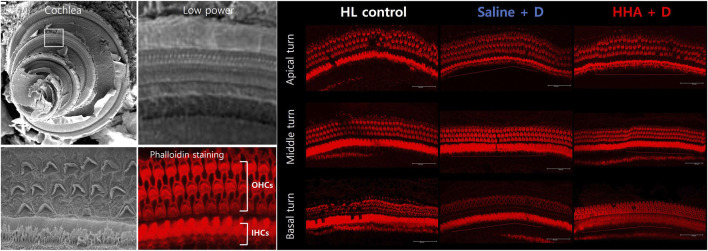
Outer hair cells in the cochlea. Using confocal microscopy, the number of lost hair cells was quantified. The number of surviving outer hair cells was consistent across the three groups.

The phalloidin-stained hair cells across the apical turn, middle turn, and basal turn are illustrated in Figure 5. For the apical turn, the counts of surviving outer hair cells per 200 µm were 74 ± 2 for the HL control group, 75 ± 2 for the saline + D group, and 75 ± 3 for the HHA + D group. In the middle turn, the counts were 73 ± 2, 73 ± 4, and 73 ± 3, respectively. For the basal turn, the numbers were 73 ± 2, 75 ± 4, and 73 ± 4, respectively. A quantitative analysis was conducted on the numbers of inner and outer hair cells (Supplementary Figure S6). The count of surviving outer hair cells remained consistent across all three groups. When comparing the quantitative analysis results of outer hair cells between the HHA + D group and the conventional HA + D group, there was a slight increase in outer hair cells in the HHA + D group across the three turns, although the difference was not statistically significant (Supplementary Figure S4).

### ABR results for hearing threshold

**FIGURE 6:**
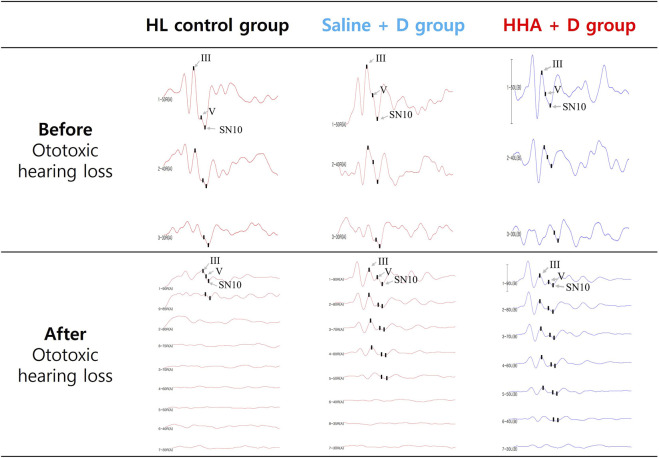
Auditory brainstem responses (ABR) waveforms displayed the III/V wave ratio and SN10 in three groups. Before the administration of the drug, hearing thresholds for all three groups ranged from 20 to 35 dB SPL. Following the induction of hearing loss and the injection of the drug, no improvement in auditory function was observed in the control group.

The ABR waveforms were interpreted as previously described, and representative waveforms, as well as the III/V wave ratio and SN10, were illustrated in Figure 6.

After ototoxic hearing loss, hearing thresholds deteriorated to a range of 68–73 dB SPL across all three groups. By day 60, the mean improvement in hearing at 8 kHz (Δ threshold relative to the poorest hearing threshold) was 20 ± 14 dB SPL for the HHA + D group and 15 ± 11 dB SPL for the saline + D group. This translates to a 5 dB SPL greater mean hearing improvement in the HHA + D group compared to the saline + D group, although the difference was not statistically significant (*p* = 0.483). At frequencies of 16 and 32 kHz, the mean hearing improvements were 23 ± 17 dB SPL and 14 ± 11 dB SPL for the HHA + D group, and 15 ± 13 dB SPL and 20 ± 15 dB SPL for the saline + D group, respectively. At 16 kHz, the HHA + D group demonstrated a 7 dB SPL greater mean hearing improvement than the saline + D group; however, this was not statistically significant (*p* = 0.158).

**FIGURE 7:**
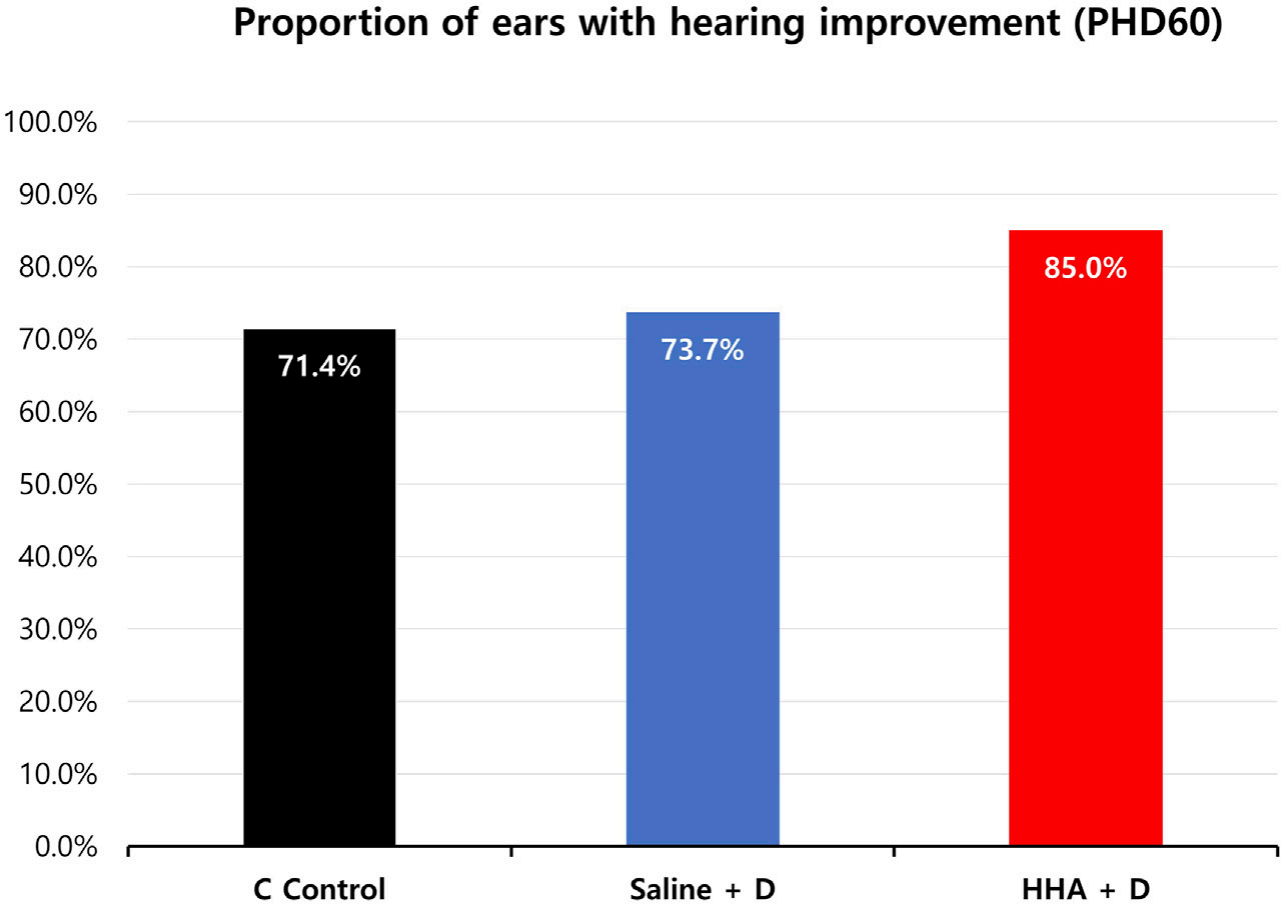
Percentage of ears showing clinically significant hearing improvement (≥15 dB SPL) at least one frequency. In the HHA + D group, clinically significant improvement was observed in 85.0% of ears, compared to 73.7% in the saline + D group and 71.4% in the HL control group.

In terms of clinically significant improvement across at least one frequency, the proportions were 85.0% for the HHA + D group, 73.7% for the saline + D group, and 71.4% for the HL control group (Figure 7). And the hearing improvement rate in the previously conducted Conventional HA + D group was 63.6% (Supplementary Figure S5). The HHA + D group had the highest proportion of ears with clinically significant improvement.

### HPLC analysis of perilymph

**FIGURE 8:**
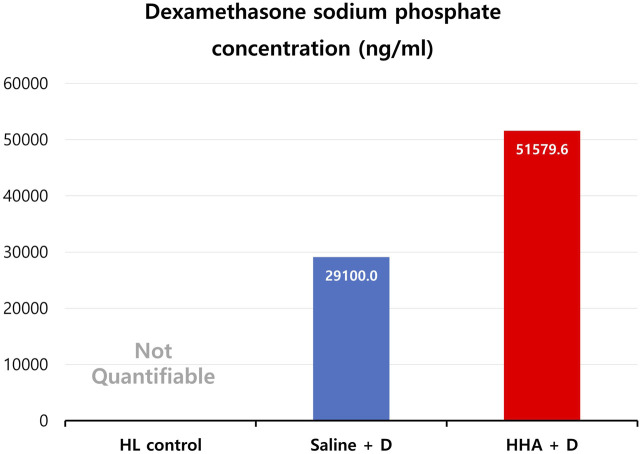
Dexamethasone (D) phosphate concentration in cochlear perilymph. While D was not detected in the HL control group (non-quantifiable due to its extremely low concentration), concentrations of 29 ± 2 μg/mL and 52 ± 16 μg/mL (mean ± SE) were identified in the saline + D and HHA + D groups, respectively.

D was undetectable in the HL control group (essentially immeasurable due to an extremely low concentration, approaching 0 ng/mL). The observed D concentrations stood at 29 ± 2 μg/mL for the saline + D group and 52 ± 16 μg/mL for the HHA + D group (mean ± standard error (SE)) (Figure 8). The D phosphate concentration in the cochlear perilymph was about 1.8 times higher in the HHA group than in the saline + D group.

## Discussion

HHA successfully met the criteria necessary for an optimal vehicle for IT drug delivery. The residence time for this agent was about 5 weeks longer, and the concentration of D phosphate in the cochlea was twice as high compared to the saline + D group. It exhibited outstanding biocompatibility with no adverse effects, and its biodegradability was appropriate. Administering HHA through a 24G needle posed no challenges. Given these benefits, we anticipate this innovative formulation of HHA will pave the way for a new IT drug-delivery vehicle specifically for acute hearing loss.

Compared to other IT vehicles documented in prior studies, the residence time of HHA is remarkably prolonged. Several drug delivery vehicles have been explored to extend the middle ear residence time and enhance the delivery concentration to the inner ear. Notable examples include gelatin, poloxamer, chitosan, collagen, and click-cross-linked HA, all of which offer certain advantages (Gausterer et al., 2020; Li et al., 2020; Park et al., 2020; Cho et al., 2021; Le et al., 2023; Lei et al., 2023). However, most of these agents had residence times ranging of a mere 1–21 days. Specifically, Poloxamer 407 had a duration of 1 day (Le et al., 2023), chitosan glycerophosphate persisted for 21 days (Yu et al., 2022), and traditional HA lasted for 2 days (Park et al., 2020). In the present study, the presence of HHA near the oval and round windows was discernible for up to 45 days without being evacuated through the eustachian tube. The high viscosity and adhesiveness of HHA appear to enable the drug/vehicle to adhere effectively to the narrow furrows or grooves within the middle ear. The minute gaps between the ossicles and the round window niche might serve as crucial anatomical sites where HHA remains for extended durations, subsequently acting as a reservoir for D. Consequently, the concentration of D phosphate in the cochlear perilymph was about double that of the saline + D group. In previous research, the concentration of gadolinium was also observed to be 1.9–2.1 times higher in the HHA group compared to the saline group (Hwang et al., 2021). We speculate that the elevated drug concentration in the cochlea is attributable to the extended residence of the HHA vehicle within the middle ear.

For medical application, HHA demonstrated no adverse effects on all tested ears. This was rigorously verified through endoscopy, micro-CT, and histology. Given that the TM is extremely thin and susceptible to inflammation, certain vehicles deemed biocompatible may nonetheless elicit adverse reactions in the middle ear (Cho et al., 2021). With HHA, this was not the case: there were no instances of inflammation, infection, or lasting perforation of the TM. Moreover, HHA fully biodegraded without leaving discernible residue within 60 days. Some substances, such as methoxy polyethylene glycol-*b*-polycaprolactone block copolymer, do not degrade even after weeks, compromising the middle ear’s aptness for aeration and ossicular movement (Park et al., 2020). While Saline + D also vanished entirely within the trial period, its disappearance should be regarded more as rapid loss or drainage rather than genuine biodegradation.

Regrettably, the therapeutic benefit of HHA on hearing thresholds and cochlear hair cell counts was modest. Compared to the saline + D group, the hearing outcome was improved by 7.5 dB SPL in the HHA + D group (at 16 kHz), but there was notable difference in the number of surviving hair cells between the groups (73.0 vs. 72.9 per 200 µm). This limited effect might stem from the minimal ototoxic damage present in our animal model. The severity of hearing and hair cell loss was not pronounced, even in the HL control group: 71% of ears showcased significant hearing improvement despite no interventions, and the count of viable hair cells was nearly typical (73.2 per 200 µm). The therapeutic effect could have hit a plateau due to the initially mild ototoxic damage. Inducing more pronounced ototoxic hearing loss could potentially lead to larger disparities between the groups. For instance, in an earlier work, the ducal viscosity HA + D group outperformed the saline + D group by 10–30 dB SPL (Li et al., 2021). The quantity of live hair cells was also considerably higher in the ducal viscosity HA + D group (56.4 vs. 35.9 hair cells per 200 µm). Such pronounced therapeutic effects were observed due to the intensified ototoxic damage: hearing thresholds were at 70 dB SPL, and only 0.2–1.4 surviving hair cells per 200 µm were detected in the HL control group. However, such intense ototoxic damage is not clinically pertinent. Patients undergoing treatments such as chemotherapy or antibacterial therapy typically do not confront such acute hearing deterioration. The animal model in this study, showcasing moderate hearing loss, more closely mirrors clinical scenarios. From this research, it can be inferred that HHA + D may offer marginal therapeutic benefits even when the extent of ototoxic hearing loss is mild.

A drug or vehicle that persists for several weeks in the middle ear can help negate the need for multiple IT injections. Clinical practice guidelines indicate that as many as four injections (1–2 injections weekly) may be required to attain adequate hearing results (Chandrasekhar et al., 2019). Such repeated IT injections result in more frequent hospital visits, escalated medical costs, and repeated discomfort during the procedure. Moreover, the risk for acute otitis media and/or chronic perforation rises with each puncturing of the TM. A 1% chance of acute infection following an IT D injection has been documented (Kim et al., 2022). Given that HHA can remain in the middle ear for 5 weeks or more, it might be feasible to substitute the current standard-of-care protocol (4 consecutive IT injections over 4 weeks) with just one injection. Even if the therapeutic results are equivalent, a solitary injection with HHA would be more desirable than four separate injections using saline. Another advantage of this extended-duration IT vehicle is its potential use for proactive prevention. In many chemotherapy cases, the onset and duration of ototoxicity are predictable. Similar to our animal study, it might be possible to pretreat cancer patients ahead of a typical chemotherapy course (1–4 weeks) to enhance hearing outcomes.

This study had several limitations. First, the animal sample size was inadequate. With a more substantial animal cohort, the subtle differences observed between groups might have achieved statistical significance. The excellent biocompatibility of HHA also needs validation in a larger population to detect rare adverse events. Second, the HPLC study was not carried out on rats. Given the ear’s varied anatomy and size across species, residence time, hearing results, and cochlear pharmacokinetics might differ between rats and guinea pigs. While perilymph sampling in rats is feasible, it comes with notable drawbacks. To obtain a perilymph sample in rats, an artificial hole or fracture is created in the cochlea’s lateral bony wall. Given the position and structure of the stria vascularis, breaching this wall could contaminate the perilymph with bone fragments or blood—issues not present when sampling through the guinea pig’s round window. Third, the degree of ototoxic hearing loss in this animal model was relatively minimal. Fourth, we exclusively validated D as the active compound. Further studies are necessary to determine the therapeutic efficacy of HHA as an IT vehicle for other drugs, including D palmitate, methylprednisolone, and triamcinolone.

## Data Availability

The raw data supporting the conclusions of this article will be made available by the authors, without undue reservation.

## References

[B1] Bird P. A. Begg E. J. Zhang M. Keast A. T. Murray D. P. Balkany T. J. (2007). Intratympanic versus intravenous delivery of methylprednisolone to cochlear perilymph. Otol. Neurotol. 28 (8), 1124–1130. 10.1097/MAO.0b013e31815aee21 18043438

[B2] Bird P. A. Murray D. P. Zhang M. Begg E. J. (2011). Intratympanic versus intravenous delivery of dexamethasone and dexamethasone sodium phosphate to cochlear perilymph. Otol. Neurotol. 32 (6), 933–936. 10.1097/MAO.0b013e3182255933 21725263

[B3] Chandrasekhar S. S. Tsai Do B. S. Schwartz S. R. Bontempo L. J. Faucett E. A. Finestone S. A. (2019). Clinical practice guideline: sudden hearing loss (update). Otolaryngol. Head. Neck Surg. 161 (1_Suppl. l), S1–S45. 10.1177/0194599819859885 31369359

[B4] Cho J. A. Kim B. J. Hwang Y. J. Woo S. W. Noh T. S. Suh M. W. (2021). Effect and biocompatibility of a cross-linked hyaluronic acid and polylactide-co-glycolide microcapsule vehicle in intratympanic drug delivery for treating acute acoustic trauma. Int. J. Mol. Sci. 22 (11), 5720. 10.3390/ijms22115720 34072013 PMC8198354

[B5] Cohn D. Salomon A. H. (2005). Designing biodegradable multiblock PCL/PLA thermoplastic elastomers. Biomaterials 26 (15), 2297–2305. 10.1016/j.biomaterials.2004.07.052 15585232

[B6] Dae-Jung K. Myung-Whan S. Hui L. Yu-Jung H. (2020). Controlled release formulations for treating hearing loss and method of preparing the same (South Korea Patent No. K. I. P. Office.

[B7] Fernandez R. Harrop-Jones A. Wang X. Dellamary L. LeBel C. Piu F. (2016). The sustained-exposure dexamethasone formulation OTO-104 offers effective protection against cisplatin-induced hearing loss. Audiol. Neurootol 21 (1), 22–29. 10.1159/000441833 26789647

[B8] Garduno-Anaya M. A. Couthino De Toledo H. Hinojosa-Gonzalez R. Pane-Pianese C. Rios-Castaneda L. C. (2005). Dexamethasone inner ear perfusion by intratympanic injection in unilateral Meniere’s disease: a two-year prospective, placebo-controlled, double-blind, randomized trial. Otolaryngol. Head. Neck Surg. 133 (2), 285–294. 10.1016/j.otohns.2005.05.010 16087029

[B9] Gausterer J. C. Saidov N. Ahmadi N. Zhu C. Wirth M. Reznicek G. (2020). Intratympanic application of poloxamer 407 hydrogels results in sustained N-acetylcysteine delivery to the inner ear. Eur. J. Pharm. Biopharm. 150, 143–155. 10.1016/j.ejpb.2020.03.005 32173603

[B10] Hill G. W. Morest D. K. Parham K. (2008). Cisplatin-induced ototoxicity: effect of intratympanic dexamethasone injections. Otol. Neurotol. 29 (7), 1005–1011. 10.1097/MAO.0b013e31818599d5 18716567 PMC2720789

[B11] Hwang Y. J. Park M. Park M. K. Lee J. H. Oh S. H. Suh M. W. (2021). High-molecular-weight hyaluronic acid vehicle can deliver gadolinium into the cochlea at a higher concentration for a longer duration: a 9.4-T magnetic resonance imaging study. Front. Neurol. 12, 650884. 10.3389/fneur.2021.650884 34248816 PMC8263933

[B12] Hyun H. Kim M. S. Jeong S. C. Kim Y. H. Lee S. Y. Lee H. B. (2006). Preparation of diblock copolymers consisting of methoxy poly (ethylene glycol) and poly (ε‐caprolactone)/poly (L‐lactide) and their degradation property. Polym. Eng. Sci. 46 (9), 1242–1249. 10.1002/pen.20581

[B13] Kim Y. H. Lee D. Y. Lee D. H. Oh S. (2022). Tympanic membrane perforation after intratympanic steroid injection: a systematic review and meta-analysis. Otolaryngol. Head. Neck Surg. 166 (2), 249–259. 10.1177/01945998211012300 34058895

[B14] Le T. P. Yu Y. Cho I. S. Suh E. Y. Kwon H. C. Shin S. A. (2023). Injectable poloxamer hydrogel formulations for intratympanic delivery of dexamethasone. J. Korean Med. Sci. 38 (17), e135. 10.3346/jkms.2023.38.e135 37128878 PMC10151621

[B15] Lei X. Yin X. Hu L. Du X. Sun C. (2023). Delivery of dexamethasone to the round window niche by saturated gelatin sponge for refractory sudden sensorineural hearing loss: a preliminary study. Otol. Neurotol. 44 (2), e63–e67. 10.1097/MAO.0000000000003769 36624588

[B16] Li H. Suh M. W. Oh S. H. (2021). Dual viscosity mixture vehicle for intratympanic dexamethasone delivery can block ototoxic hearing loss. Front. Pharmacol. 12, 701002. 10.3389/fphar.2021.701002 34776942 PMC8581269

[B17] Li X. Wang Y. Xu F. Zhang F. Xu Y. Tang L. (2020). Artemisinin loaded mPEG-PCL nanoparticle based photosensitive gelatin methacrylate hydrogels for the treatment of gentamicin induced hearing loss. Int. J. Nanomedicine 15, 4591–4606. 10.2147/IJN.S245188 32612358 PMC7323796

[B18] McCall A. A. Swan E. E. Borenstein J. T. Sewell W. F. Kujawa S. G. McKenna M. J. (2010). Drug delivery for treatment of inner ear disease: current state of knowledge. Ear Hear 31 (2), 156–165. 10.1097/AUD.0b013e3181c351f2 19952751 PMC2836414

[B19] Min K. H. Rhee C. K. Jung J. Y. Suh M. W. (2012). Characteristics of adverse effects when using high dose short term steroid regimen. Korean J. Audiol. 16 (2), 65–70. 10.7874/kja.2012.16.2.65 24653873 PMC3936564

[B20] Park M. Hwang Y. J. Noh T. S. Woo S. W. Park J. H. Park S. H. (2020). Biocompatibility and Therapeutic Effect of 3 intra-tympanic drug delivery vehicles in acute acoustic trauma. Audiol. Neurootol 25 (6), 291–296. 10.1159/000506535 32403103

[B21] Seggas I. Koltsidopoulos P. Bibas A. Tzonou A. Sismanis A. (2011). Intratympanic steroid therapy for sudden hearing loss: a review of the literature. Otol. Neurotol. 32 (1), 29–35. 10.1097/mao.0b013e3181f7aba3 21192346

[B22] Shim H. J. (2016). Intratympanic steroid injection in tinnitus management. Hanyang Med. Rev. 36 (2), 125–130. 10.7599/hmr.2016.36.2.125

[B23] Wang H. Zhao Z. Chen S. (2023). Local vs. systemic use of steroids for sudden deafness with diabetes: a Systematic review and meta-analysis. Ear Nose Throat J., 014556132311700. 10.1177/01455613231170090 37039340

[B24] Yu Y. Kim D. H. Suh E. Y. Jeong S. H. Kwon H. C. Le T. P. (2022). Injectable glycol chitosan thermogel formulation for efficient inner ear drug delivery. Carbohydr. Polym. 278, 118969. 10.1016/j.carbpol.2021.118969 34973784

[B25] Zhang M. Moore G. A. Jensen B. P. Begg E. J. Bird P. A. (2011). Determination of dexamethasone and dexamethasone sodium phosphate in human plasma and cochlear perilymph by liquid chromatography/tandem mass spectrometry. J. Chromatogr. B Anal. Technol. Biomed. Life Sci. 879 (1), 17–24. 10.1016/j.jchromb.2010.11.003 21112257

[B26] Zhao Z. Yang L. Hu Y. He Y. Wei J. Li S. (2007). Enzymatic degradation of block copolymers obtained by sequential ring opening polymerization of l-lactide and ɛ-caprolactone. Polym. Degrad. Stab. 92 (10), 1769–1777. 10.1016/j.polymdegradstab.2007.07.012

[B27] Zhou Y. Zheng G. Zheng H. Zhou R. Zhu X. Zhang Q. (2013). Primary observation of early transtympanic steroid injection in patients with delayed treatment of noise-induced hearing loss. Audiol. Neurootol 18 (2), 89–94. 10.1159/000345208 23208457

