# Supplementary figures and images for: Biosafety and potency of high-molecular-weight hyaluronic acid with intratympanic dexamethasone delivery for acute hearing loss
Source: Front Pharmacol. 2024 Jan 16;15:1294657. doi: 10.3389/fphar.2024.1294657 (PMC10824912; doi:10.3389/fphar.2024.1294657)

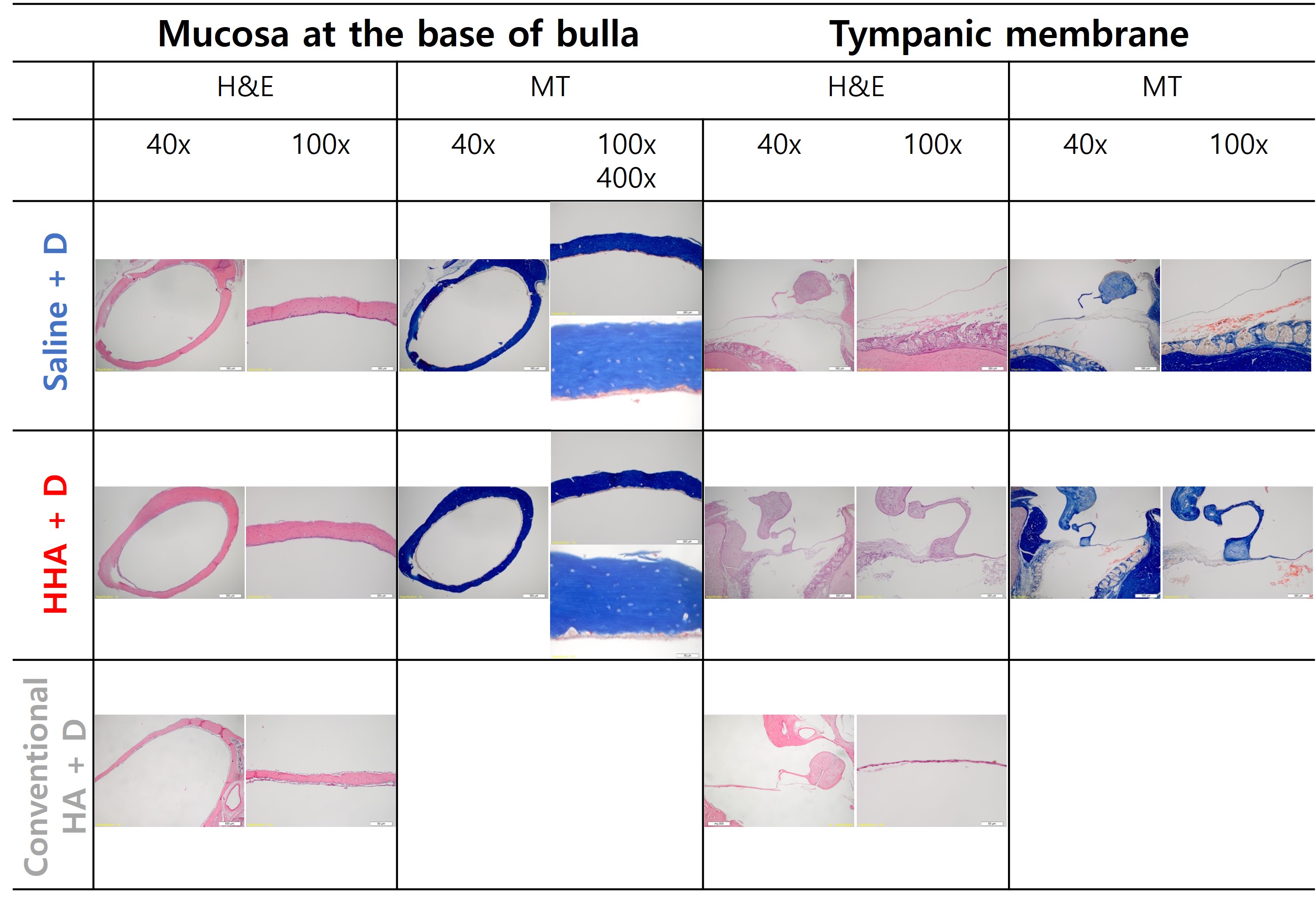

Supplement: Supplementary file 1 [file Image3.JPEG]

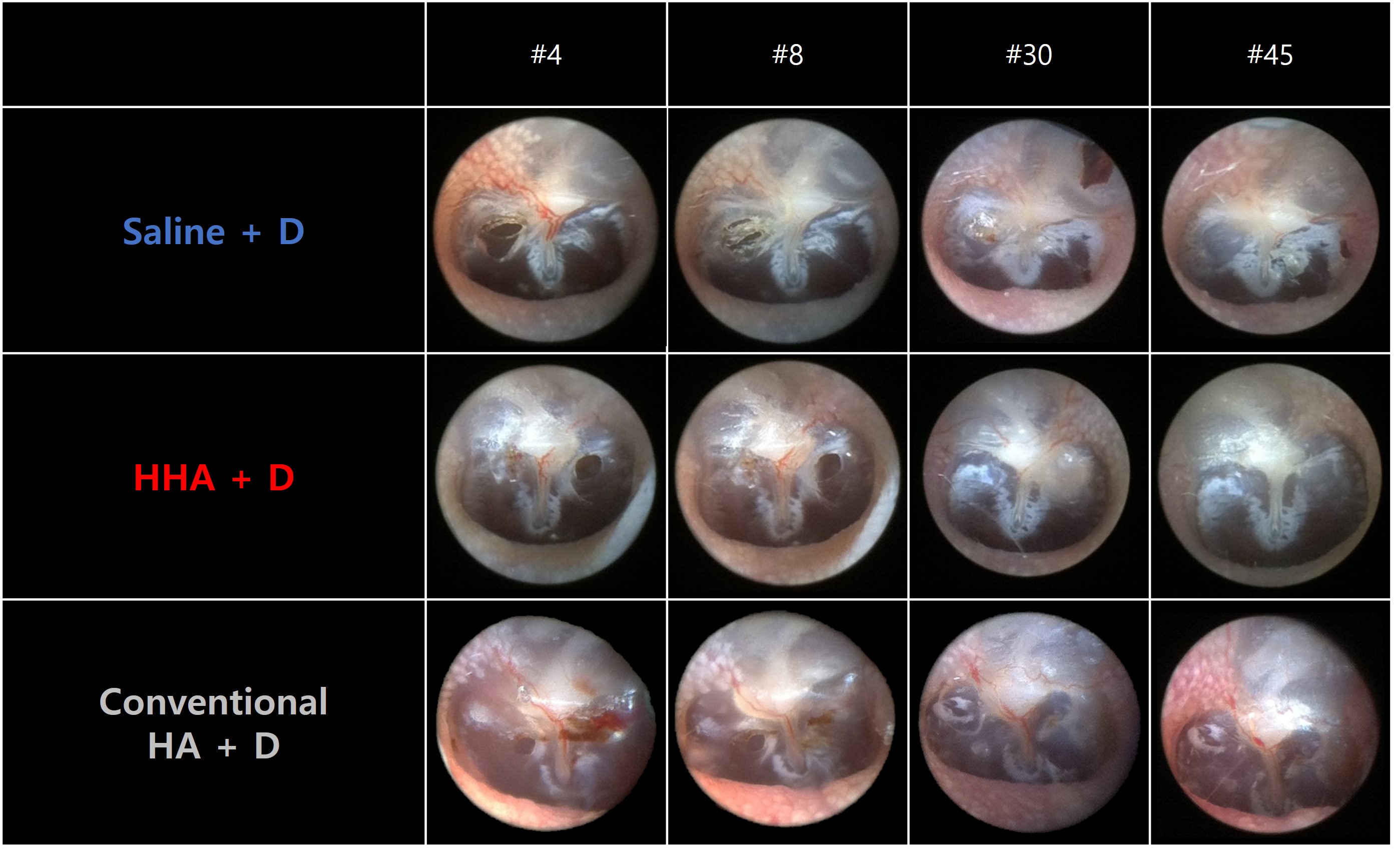

Supplement: Supplementary file 2 [file Image1.JPEG]

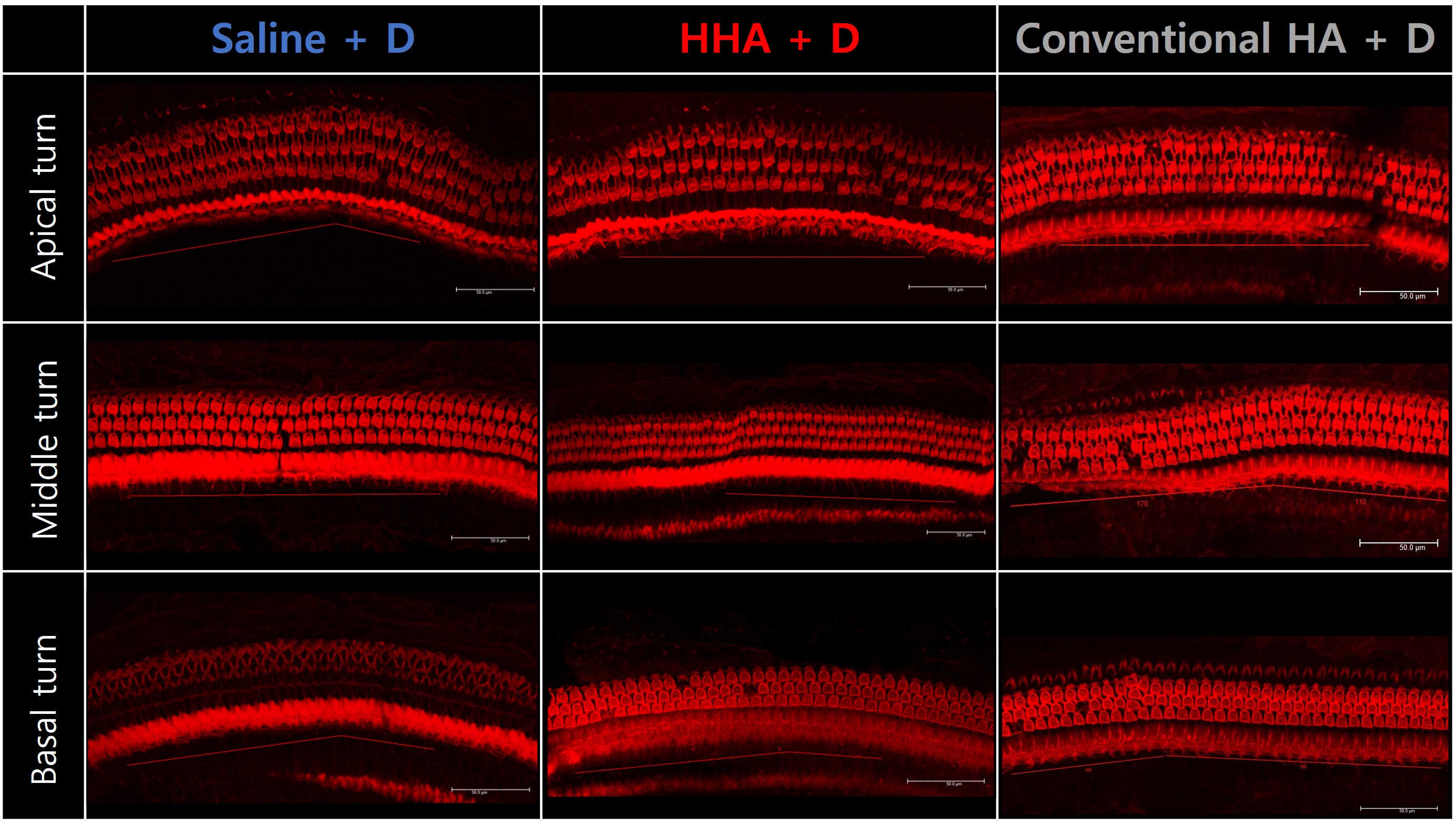

Supplement: Supplementary file 3 [file Image4.JPEG]

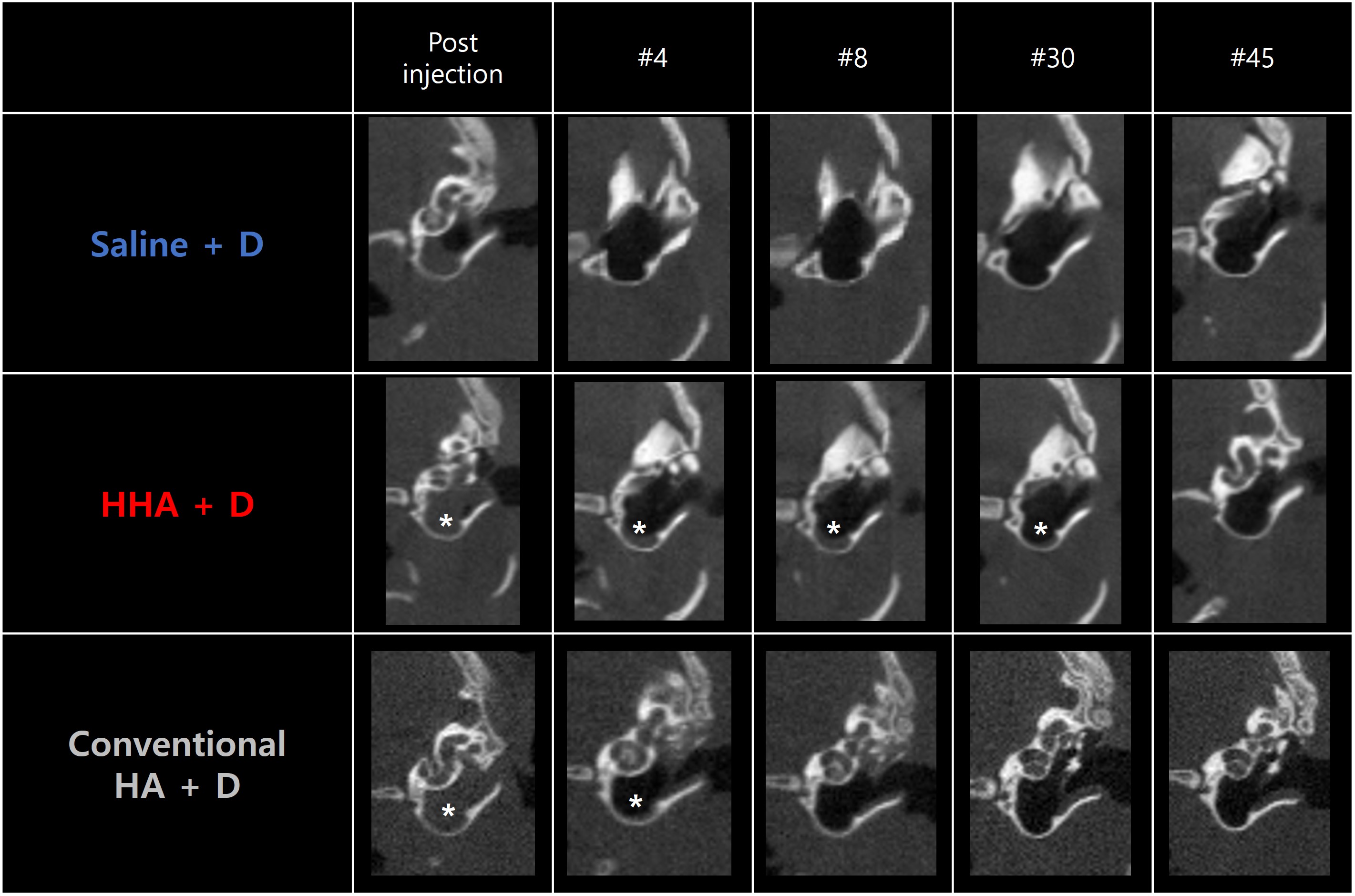

Supplement: Supplementary file 4 [file Image2.JPEG]

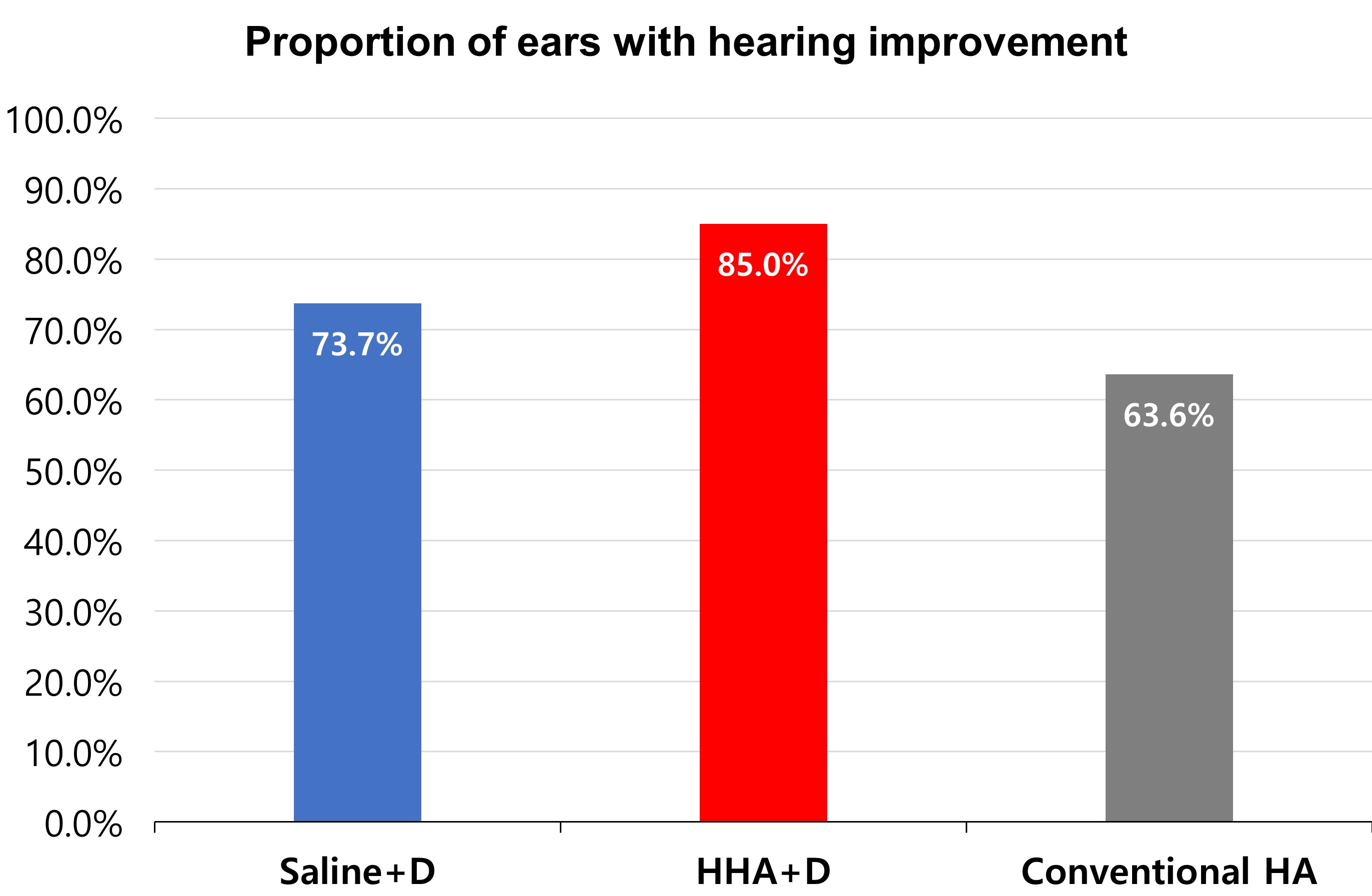

Supplement: Supplementary file 5 [file Image5.JPEG]

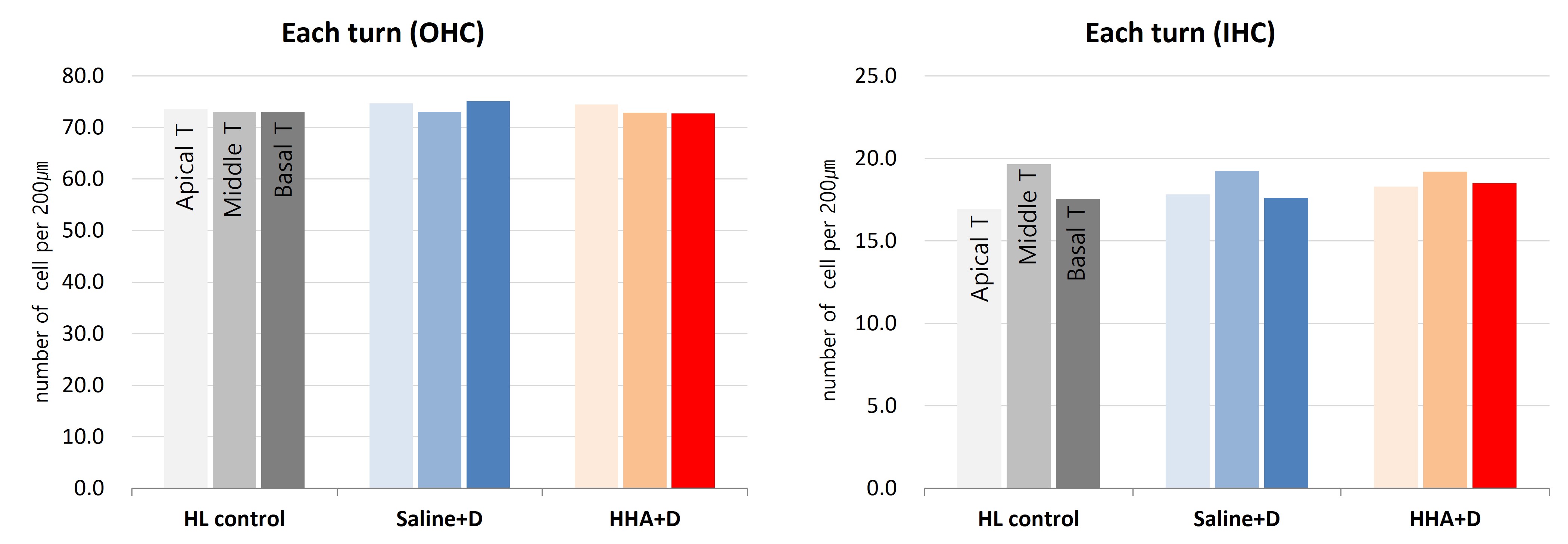

Supplement: Supplementary file 7 [file Image6.JPEG]
