# Supplementary material for: Biosafety and potency of high-molecular-weight hyaluronic acid with intratympanic dexamethasone delivery for acute hearing loss
Source: Front Pharmacol. 2024 Jan 16;15:1294657. doi: 10.3389/fphar.2024.1294657 (PMC10824912; doi:10.3389/fphar.2024.1294657)
Supplement: Supplementary file 6 [file DataSheet1.PDF]

Supplementary figure legend

Supplementary figure 1. Endoscopic observations of the TM.

Perforations created during injection healed within 20–26 days. No animals displayed residual TM perforation after 60 days. Perforation closure was observed 22± 13 days post-IT injection in the saline + D group, 21 ± 12 days in the HHA + D group and 24.2 ± 9.3 days in the conventional HA+D group.

Supplementary figure 2. Micro-CT imaging of the bulla.

Soft tissue density, indicative of vehicle/drug presence, was identified in the bulla. Persistence of this signal was monitored. The duration of vehicle/drug persistence in the bulla was 1 ± 0 days in the saline + D group and 1.8 ± 2.4 days in the conventional HA+D group whereas in the HHA + D group, it persisted for 41 ± 27 days. The observed duration in the bulla was notably longer in the HHA+D group than in the conventional HA + D group. The asterisk denotes the soft tissue density signal in the CT image (indicative of residual vehicle/drug).

Supplementary figure 3. Middle ear histology.

Mucosal histology at the bulla's base showed similarity between the saline + D and HHA + D groups when stained with hematoxylin and eosin (H&E) and Masson's trichrome (MT). No signs of inflammatory cell infiltration or mucosal thickening were evident under both low-power (40×) and high-power (400×) magnification. The thickness of the tympanic membrane was consistent between groups. Collagen and fibrin composition patterns, assessed via MT staining, also exhibited no discernible differences.

Supplementary figure 4. Hair cells in the cochlea.

Using confocal microscopy, the number of lost hair cells was quantified. The number of surviving outer hair cells was consistent across the three groups. The number of hair cells quantified using the three-turn average was 74.3 ± 1.1 cells in the Saline + D group, 73.4 ± 1.0 in HHA + D group and 72.1 ± 5.9 cells in the conventional HA+D group.

Supplementary figure 5. Percentage of ears showing clinically significant hearing improvement (≥ 15 dB SPL) at least one frequency.

In the HHA + D group, clinically significant improvement was observed in 85.0% of ears, compared to 73.7% in the saline + D group and 63.6% in the conventional HA + D group.

Supplementary figure 6. cell count of outer and inner hair cell.

Outer hair cell count of whole turn is 219.6 ± 0.3 in control group, 222.8 ± 1.1 in the saline + D group and 220.1 ± 1.0 in the HHA + D group. And Inner hair cell count of whole turn is 54.1 ± 1.4 in control group, 54.7 ± 0.9 in the saline + D group and 56.0 ± 0.5 in the HHA + D group. There was no statistically significant difference between the inner hair cells and outer hair cells between the three groups.
